# Correction to: A multicenter randomized trial to assess the efficacy of CONvalescent plasma therapy in patients with Invasive COVID-19 and acute respiratory failure treated with mechanical ventilation: the CONFIDENT trial protocol

**DOI:** 10.1186/s12890-021-01574-8

**Published:** 2021-07-26

**Authors:** Benoît Misset, Eric Hoste, Anne Françoise Donneau, David Grimaldi, Geert Meyfroidt, Michel Moutschen, Veerle Compernolle, André Gothot, Daniel Desmecht, Mutien Garigliany, Tome Najdovski, Pierre François Laterre

**Affiliations:** 1 Department of Intensive Care Medicine, Liege University Hospital, Domaine Universitaire du Sart Tilman, 4020 Liège, Belgium; 2 Department of Intensive Care Medicine, Gent University Hospital, Gent, Belgium; 3 Biostatistic Unit, Public Health Department, Liège University, Liège, Belgium; 4 Department of Intensive Care Medicine, Cliniques Universitaires de Bruxelles - Erasme, Université Libre de Bruxelles, Brussels, Belgium; 5 Department of Intensive Care Medicine, University Hospitals Leuven, Leuven, Belgium; 6 Department of Infectious Diseases, Liege University Hospital, Liège, Belgium; 7 Blood Services from the Red Cross, Mechelen, Belgium; 8 Department of Immunohematology, Liege University Hospital, Liège, Belgium; 9 Department of Animal Pathology, Liège University, Liège, Belgium; 10 Blood Services From the Red Cross, Suarlée, Belgium; 11 Department of Intensive Care Medicine, Saint-Luc University Hospital, Brussels, Belgium

## Correction to: BMC Pulm Med (2020) 20:317 10.1186/s12890-020-01361-x

Following publication of the original article [1], it was brought to the authors’ attention that sentences in the method section were not appropriate, due to an omission in the update of a prior version.

Namely, the following sentences have been deleted in the corrected version:

*“Specifically, the interim assessment of the SOC group will be based on the numbers of patients and of deaths shown in Table 2. The recommendation will be made to stop accruing patients in the SOC group if the number of deaths observed in this group suggests a true mortality rate in excess of 0.4. The rule for DSMB recommendation shown in Table 2 will not be binding.”*

*Table 2* has been removed, and *table 3* has been re-named as “*Table 2*” in the corrected body text. The error has since been corrected in the original article.

Similarly, in the electronic supplement,

- the following sentences have been deleted:

*“Specifically, the interim assessment of the SOC group will be based on the numbers of patients and of deaths shown in Table 1. The recommendation will be made to stop accruing patients in the SOC group if the number of deaths observed in this group suggests a true mortality rate in excess of 0.4. The rule for DSMB recommendation shown in Table 1 will not be binding.”*

- *Table 1* has been removed, and *Table 2* has been re-named as “*Table 1”* in the corrected body text.
- the word “*three*” has been replaced by “*two*” in the following sentence, (paragraph 9.10):

“Because of the considerable uncertainties about the outcomes that will be observed in the two arms of the trial, the DSMB will be allowed to recommend design changes based on the observed data at the times of the interim analyses.”

The authors apologize for any inconvenience caused.
